# Development of a rapid reverse genetics system for feline coronavirus based on TAR cloning in yeast

**DOI:** 10.3389/fmicb.2023.1141101

**Published:** 2023-03-23

**Authors:** Hongmin Cao, Haorong Gu, Hongtao Kang, Honglin Jia

**Affiliations:** State Key Laboratory of Veterinary Biotechnology, Harbin Veterinary Research Institute, Chinese Academy of Agricultural Sciences, Harbin, China

**Keywords:** FCoV, FIPV, reverse genetics, transformation-associated recombination, yeast

## Abstract

**Introduction:**

Reverse genetics has become an indispensable tool to gain insight into the pathogenesis of viruses and the development of vaccines. The yeast-based synthetic genomics platform has demonstrated the novel capabilities to genetically reconstruct different viruses.

**Methods:**

In this study, a transformation-associated recombination (TAR) system in yeast was used to rapidly rescue different strains of feline infectious peritonitis virus, which causes a deadly disease of cats for which there is no effective vaccine.

**Results and discussion:**

Using this system, the viruses could be rescued rapidly and stably without multiple cloning steps. Considering its speed and ease of manipulation in virus genome assembly, the reverse genetics system developed in this study will facilitate the research of the feline coronaviruses pathogenetic mechanism and the vaccine development.

## 1. Introduction

Feline infectious peritonitis (FIP), caused by feline infectious peritonitis virus (FIPV), is a severe and lethal systemic infection of cats. Clinically, FIP can be divided into exudate type and nonexudate type based on whether or not peritoneal effusion is observed. Once exudative FIP symptoms appear, the mortality rate can be as high as 100%. Currently, there is still no effective vaccine available for this deadly disease. Accurate diagnosis of this disease is also difficult due to poor understanding of the pathogenetic mechanisms of FIPV. There are two pathogenic types of feline coronaviruses (FCoV): feline enteric coronavirus (FECV) and feline infectious peritoniitis virus (FIPV). The prevalence of FCoV infection in feline populations is generally high and may exceed 90% in multicat environments. The incidence of FIP, however, is very low and rarely affects more than 5% of infected cats (Pedersen, 2009; Drechsler et al., 2011). FECV is generally considered a mild, nonpathogenic form of FCoV. Oral FECV infection either is subclinical or causes very mild, nonspecific clinical symptoms, such as transient anorexia, in older cats (Vogel et al., 2010). However, in young (Specific Pathogen Free) SPF kittens, oral FECV infection causes severe enteritis (Pedersen et al., 1981; Addie and Jarrett, 1992).

FCoV belong to the genus *Alphacoronavirus* within the family *Coronaviridae* of the order *Nidovirales* (Tekes and Thiel, 2016). FCoV is single-stranded, positive-sense RNA virus. Their genomes are about 28 kb in size and encode at least 11 open reading frames (ORFs), with two of the major ORFs encoding a replicase. ORF1a and ORF1b encode 16 nonstructural proteins (NSPs); four ORFs encode structural proteins (spike protein S, envelope protein E, membrane protein M, and nucleocapsid protein N); and five ORFs encode the nonstructural proteins 3a, 3b, 3c, 7a, and 7b (Ziebuhr et al., 2000; Dye and Siddell, 2005; Tekes et al., 2008). It is believed that FIPV evolved from FECV. Based on extensive sequence analysis of FECV and FIPV isolates, it was believed that mutations in S and accessory genes are involved in the development of FIP (Herrewegh et al., 1995; Vennema et al., 1998; Kennedy et al., 2001; Pedersen et al., 2009; Chang et al., 2012; Licitra et al., 2013; Bank-Wolf et al., 2014; Lewis et al., 2015). However, substituting FIPV S gene into the FECV skeleton did not induce FIP biotype transformation (Ehmann et al., 2018; Wang et al., 2021). Therefore, the pathogenic mechanisms it has evolved that distinguish from FECV is still unclear. FCoVs can be divided into serotypes I and II according to the spike gene. Both FECV and FIPV contains the two serotypes. There is consistent evidence indicating that type II FCoVs were evolved from the recombination of partial RNA sequences containing the speak genes of type I FCoVs and canine coronavirus (CCoVs; Herrewegh et al., 1998; Shiba et al., 2007; Terada et al., 2014). Although type II FCoV is easily propagated in cell culture, type I FCoV isolates generally grow poorly in cell culture. The vast majority (70 to 98%) of natural infections occurring worldwide are caused by serotype I FCoVs, which serotype II FCoVs are less common (Hohdatsu et al., 1992; Addie et al., 2003; Benetka et al., 2004; Kummrow et al., 2005; Shiba et al., 2007; Duarte et al., 2009; Lin et al., 2009; Amer et al., 2012; Li et al., 2019).

Currently, reverse genetic systems for coronaviruses have been developed based on various cloning platforms including bacterial artificial chromosome (BAC) vectors (Almazan et al., 2000), vaccinia virus vector (Thiel and Siddell, 2005), transformation-associated recombination (TAR) system in yeast (Thao et al., 2020), circular polymerase extension reaction strategy (CPER; Torii et al., 2021), and infectious subgenomic amplicons (ISA) (Melade et al., 2022). The successful establishment of reverse genetics systems for FCoVs based on BAC vector (Balint et al., 2012) and vaccinia virus (Ehmann et al., 2018) have also been reported. However, coronaviruses are often difficult to clone and manipulate in Escherichia coli due to the large size of the genomes. Meanwhile, occasional instability occurred in the genomes assembled by the BAC vector (Almazan et al., 2000; Yount et al., 2000). The major drawback of the vaccinia virus-based rescuing system is that it required multiple cloning steps, and inconvenient screening processes. Although infectious viruses could be generated by the ISA method, viral populations rescued through this system are often more diverse than that derived from an infectious clone (Mohamed Ali et al., 2018; Driouich et al., 2019). Therefore, generation of a more infectious clone is still necessary for studying the pathogenic mechanism of FIPV. In this study, we have further improved yeast-based TAR system by shorting the construction steps, and the construction of infective cDNA of FCoV could be completed in 1 week. Overall, we provide a rapid, reverse genetics system for assembling an infectious clone of FCoV *via* a yeast-based TAR which would benefit for the FCoV vaccine development and pathogenetic mechanism research.

## 2. Materials and methods

### 2.1. Cells and virus general culture conditions

Crandell-Rees feline kidney (CRFK) cells were available in our laboratory. Baby Hamster Kidney cell clone (BSR-T7/5), which stably expresses T7 RNA polymerase (T7 pol), was kindly provided by Zhigao Bu (Harbin Veterinary Research Institute, Chinese Academy of Agricultural Sciences). Felis catus whole-fetus 4 (FCWF-4) cells were purchased from the ATCC. All cells were maintained in Dulbecco’s modified Eagle’s medium (DMEM; Sigma Aldrich, D6429) with 10% FBS (Clark, FB25015) at 37°C in an atmosphere of humidified air containing 5% (v/v) CO_2_.

### 2.2. Bacterial and yeast strains

TransforMax Epi300 cells (Lucigen) were used to propagate the plasmid. All yeast transformation experiments were performed using *Saccharomyces cerevisiae* VL6-48 N (MATα trp1-Δ1 ura3-Δ1 ade2-101 his3-Δ200 lys2 met14 cir°), which was provided by Vladimir Larionov (Laboratory of Biosystems and Cancer, National Cancer Institute, Bethesda, Maryland, 20,892, USA; Wach et al., 1994; Kouprina et al., 1998; Noskov et al., 2002). Yeast cells were first cultured in YPDA broth (Takara Bio, 630,306), then transformed cells were placed on a minimum synthetic definition (SD) agar without histidine (SD-His; Takara Bio, Clontech, 630,415,630,411).

### 2.3. Virus strains

Full-length nucleotide (nt) sequences of FIPV I Black, FIPV II DF-2, and FECV I MG893511 (GenBank accession numbers EU186072.1, JQ408981.1, and MG893511.1, respectively) were independently used for the construction of each infectious cDNA clone using the TAR system. The FIPV II DF-2 were purchased from the ATCC. FIPV Black is a culture-adapted serotype virus (Black, 1980). But the ability to induce Symptomatic FIP is lost in cats (Thiel et al., 2014). FIPV DF-2 is a culture-adapted type II strain and has a strong pathogenic ability to cats (Balint et al., 2014). Serotype I FECV (FECV I MG89351) cannot be propagated in standard cell culture and causes an asymptomatic but persistent infection in cats (Ehmann et al., 2018).

### 2.4. Viral genome assembly in yeast

The plasmid of pCC1BAC-His3 was used for TAR cloning. The sequence of yeast centromere (CEN6), yeast replication origin (autonomously replicating sequence (ARS)), and yeast selectable marker (His3) shown in Supplementary Table 3 were synthesized by Comate Bioscience Company Limited (Gibson et al., 2010) and cloned into the pCC1BAC vector to produce the pCC1BAC-His3 vector. The cDNA fragments were amplified by PCR, using primers that overlapped at least 50 bp with segments containing the 5′ or 3′ ends of different viral genomes (Supplementary Table 1). Amplification was performed using KOD DNA polymerase (TOYOBO, KOD-401) according to the manufacturer’s instructions. The template used to generate the cloned TAR fragment is shown in Table 1.

**Table 1:** Cloning RNA virus genome using synthetic genomics platform.

Virus	Size (kb)	Template	Fragment generation	Number of fragments	Virus rescue
rT7-Black-S_DF-2_	29.2	Synthetic DNA plasmid	PCR	8	Yes
rCMV-Black-S_DF-2_	29.2	Synthetic DNA plasmid	PCR	9	Yes
rCMV-MG893511-S_DF-2_	32.9	Synthetic DNA plasmid	PCR	10	Yes
rCMV-Black-S	29.3	Synthetic DNA plasmid	PCR	9	Yes

Yeast conversion was performed using a high-efficiency lithium acetate/SS vector DNA/PEG method. Briefly, yeast cells were grown with agitation in a YPDA-rich medium at 30°C and activated twice until the optical density reached 1.0 at 600 nm before 3 ml of the culture was used for each transformation event. Transformation was carried out using a DNA mixture with a total concentration of 1 μg for all fragments. The following transformation yeast cells were plated on SD-His plates and incubated at 30°C for 48 h. Transformants were picked and suspended in 3 ml SD-His liquid medium and incubated overnight at 30°C, with shaking. For the preparation of crude, genomic DNA from transformants, aliquots (1 ml) of each culture were centrifuged, and the resulting cells pellets were resuspended in 200 μL TE Buffer (10 mM Tris–HCl, 1 mM EDTA. pH 8.0 [Amresco; Solarbio Life Sciences]) before being heated to 100°C for 10 min and then placed on an ice bath for 10 min. These genome preparations were used as templates for the identification by PCR of yeast transformants containing appropriately assembled FCoV DNA fragments using primers designed to amplify all fragment junctions (Supplementary Table 2). Clones harboring plasmids shown to contain all the expected overlapping junctions were identified, and plasmids were extracted from 20 ml cultures of those clones using the Yeast Plasmid Kit (OMEGA) according to the manufacturer’s instructions but with the following modifications: (1) buffer YP I (500 μL) was supplemented with 40 μL of enzymolysis solution (10 mg/ml enzymolysis 20-T; 50 mM Tris–HCl pH 7.5; 50% (v/v) glycerol) and 100 μL β-mercaptoethanol, and (2) the mixture was incubated at 37°C for 1 h prior to the addition of buffer YP II. Then the plasmids were purified according to the instructions.

### 2.5. Transformation of yeast plasmids into *Escherichia coli*

Purified recombinant yeast plasmids containing the appropriately assembled, full-length viral genome were transformed into *E. coli* Epi300 cells. Yeast plasmid (30 ng) was transferred into EPI300 cells. After shaking, copycontrol inducer (1:1000) was added to the bacteria and the recombinant plasmid was purified from 200 ml bacterial culture using the QIAfilter Plasmid Midi Kit (QIAGEN).

### 2.6. Recovery of viruses

T7 or CMV promoters were added to the 5′ end of the viral genome, and the hepatitis delta virus ribozyme (HDVrz) and bovine growth hormone (BGH) transcription terminal signals were added after poly (A) sequences. In short, the recombinant plasmids were transfected into BSR-T7/5 cells or CRFK cells. The rescued viruses were passaged once in CRFK cells and harvested by freezing and thawing three times. Specific-genome sequencing of the recombinant viruses was performed before viruses were titrated and stored at −80°C.

### 2.7. Virus growth kinetics

Virus titration were conducted in 96-well plates. The viruses were serially diluted tenfold, and 100 μL of each dilution was added to separate wells and incubated for 2 h at room temperature. After incubation, the serum-free culture medium was replaced with 2% (v/v) FBS DMEM culture medium, and wells containing visible cytolytic lesions were recorded. Viral titers were expressed as the median tissue culture infective dose Log10 (TCID_50_/ml) according to the method of Reed and Muënch (Pizzi, 1950). The growth kinetic of rCMV-Black-S was determined with FCWF-4 cells and the CRFK cells were used for the growth evaluation of the remaining viruses. The cells were infected with the virus at an MOI of 0.01, and the supernatant containing virus was collected and titrated at 6 h, 12 h, 24 h, 36 h, and 48 h post-infection, respectively. The growth curve of the virus was generated using GraphPad Prism 8.

### 2.8. Immunofluorescence assay

CRFK cells were mock infected or infected with rFCoV at an MOI of 0.1 for 12 h and then washed three times with PBS. After washing, CRFK cells were fixed with 4% (v/v) paraformaldehyde (Biosharp, BL302A) in PBS for 15 min and then permeabilized with 0.3% (v/v) Triton X-100 (Solarbio Life Sciences, T8200) in PBS for 15 min. After washing, the CRFK cells were incubated with mouse anti-FIPV N antibody at a dilution of 1:2, 000 at 4°C overnight. Subsequently, Alexa Fluor 488 (1;10,000)-conjugated Goat Antimouse IgG (Invitrogen, A11001) was added as a secondary antibody. After washing three times with PBS, DAPI (SIGMA, D9542-5MG) was added for 15 min. After washing three times with PBS, fluorescence was observed under an inverted fluorescence microscope (Zeiss).

### 2.9. Purification of recombinant FCoV

The cell culture supernatants (100 ml) were harvested on day 4 post-infection and centrifuged (4,000 × g for 30 min at 4°C) to remove cell debris. The recombinant FCoV were then pelleted by ultracentrifugation at 60,000 × g for 2 h at 4°C. The pellets were resuspended in Hepes-saline buffer at 4°C overnight and then purified through a 10–20-30% discontinuous sucrose gradient at 60,000 × g for 4 h at 4°C. The FCoV at the bottom of the tube was collected. The highly purified virus particles obtained were resuspended in 100 μL of Hepes-saline buffer.

### 2.10. Western blotting

For the western blot analysis, rescued viruses were mixed with 4 × SDS loading buffer and boiled for 10 min. The samples were analyzed using 12% SDS-PAGE and transferred onto a PVDF membrane. The membrane was blocked with a blocking buffer (Thermo Fisher Scientific, Waltham, USA) and incubated with a mouse anti-FIPV N antibody, at a dilution of 1:2,000, at room temperature for 2 h. After incubation, the membranes were washed five times with TBST buffer and incubated with DyLight 800-labeled anti-mouse IgG at a dilution of 1:10,000 for 1 h at room temperature.

## 3. Results

### 3.1. Design of FCoV genome assembly

**Figure 1:**
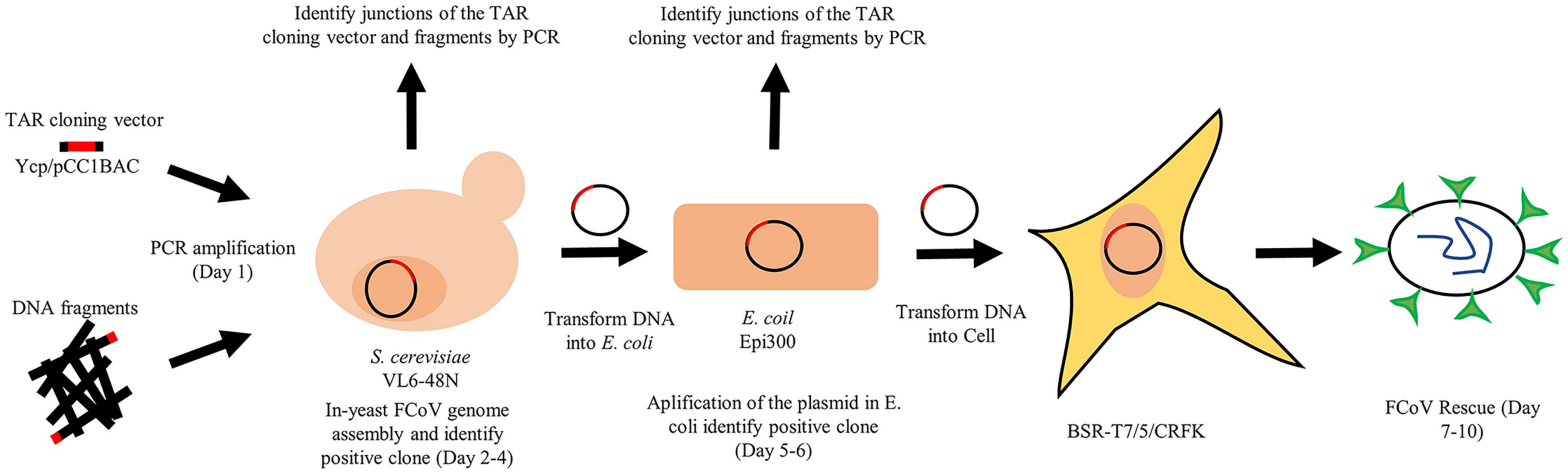
The full process of viral genome assembly and virus rescue.

The full process for constructing an infectious clone of feline coronaviruses is shown in Figure 1. The design of different FCoV fragments and the assembly of a complete genome are indicated in the individual figures. Each fragment overlaps its neighbor by at least 50 base pairs. The TAR clone vector and FCoV genomic fragments were co-transferred into yeast cells to assemble the whole virus genome and the shuttle vector, and the recombinant plasmids in yeast were transformed into *E. coli*. Finally, the plasmids were extracted from the *E. coli* to rescue recombinant viruses.

### 3.2. Rescue of an infectious FIPV Black-S_DF-2_ virus strain driven by a T7 promoter

**Figure 2:**
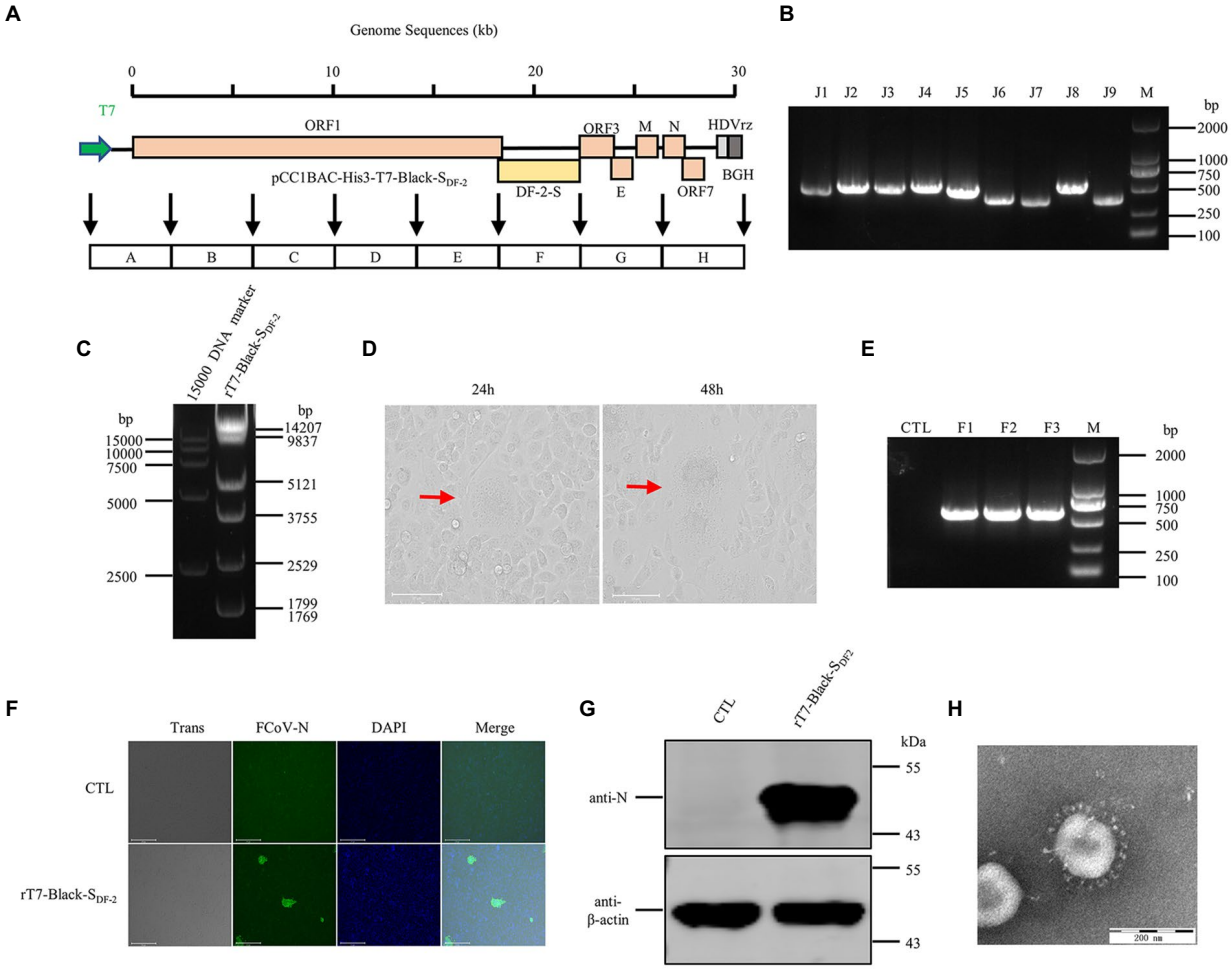
The recovery and identification of chimeric FIPV strain Black-SDF-2 driven by a T7 promoter. **(A)** Strategy diagram for the construction of pCC1BAC-His3-T7-Black-SDF-2. **(B)** Identification of fragment interfaces of the plasmids recovered from bacteria. **(C)** Enzyme digestion of pCC1BAC-His3-T7-Black-SDF-2 plasmid (NotI and NheI). **(D)** Significant cytopathic effects (CPE) were observed at 24 and 48 h after infection with CRFK cells. **(E)** PCR identification of the rescued recombinant virus. **(F)** and **(G)** rT7-Black-SDF-2 virus infected CRFK cells for 12 h was identified by IFA (Scale Bar, 275 μm) and WB. **(H)** Electron microscopy examination of the purified virus.

First, we tried to rescue a chimeric virus with a type II S protein in the FIPV Black backbone, using a T7 promoter and a stable cell line expressing T7 RNA polymerase (BSR-T7/5). The strategy diagram of constructing a full-length FIPV T7-Black-S_DF-2_ clone in the pCC1BAC-His3 vector was shown in Figure 2A. PCR was used to confirm the presence of viral genome segments and segment junctions in DNA isolated from yeast clones. Plasmids from positive clones were transferred into *E. coli*, and the presence of a plasmid harboring the entire FCoV genome was verified by PCR (Figure 2B), restriction enzyme analysis (Figure 2C), and sequencing. The results indicated that this chimeric sequence contained only one nucleotide mutation, which might have arisen as a result of DNA polymerase infidelity during the PCR amplification step. Transfection of the purified plasmid into CRFK cells resulted in the visible cytopathic effect of syncytial fusion about 24 h after transfection (Figure 2D).

The first three generations of the rescued rT7-Black-S_DF-2_ strain were extracted for reverse transcription to give cDNAs, the specific sequences of which were confirmed by PCR (Figure 2E). Expression of the N protein of rT7-Black-S_DF-2_ was verified by IFA using a monoclonal antibody. The cytoplasm of typical syncytic cells showed obvious staining of N protein (Figure 2F). Moreover, western blot analysis revealed a band that reacted with the monoclonal antibody against N protein, in rFCoV particles released by transfected cells (Figure 2G). As shown in Figure 2H, electron microscopy revealed the presence of virus particles with coronavirus-like morphological characteristics, including typical spike structures, in the supernatant of cells. Next generation sequencing verified that the sequence of the rescued virus was 100% identical to the sequence cloned in the assembled recombinant plasmid.

### 3.3. Rescue of virus strain FIPV Black-S_DF_-2, FECV MG893511-SDF-2, and FIPV Black with a CMV promoter

**Figure 3:**
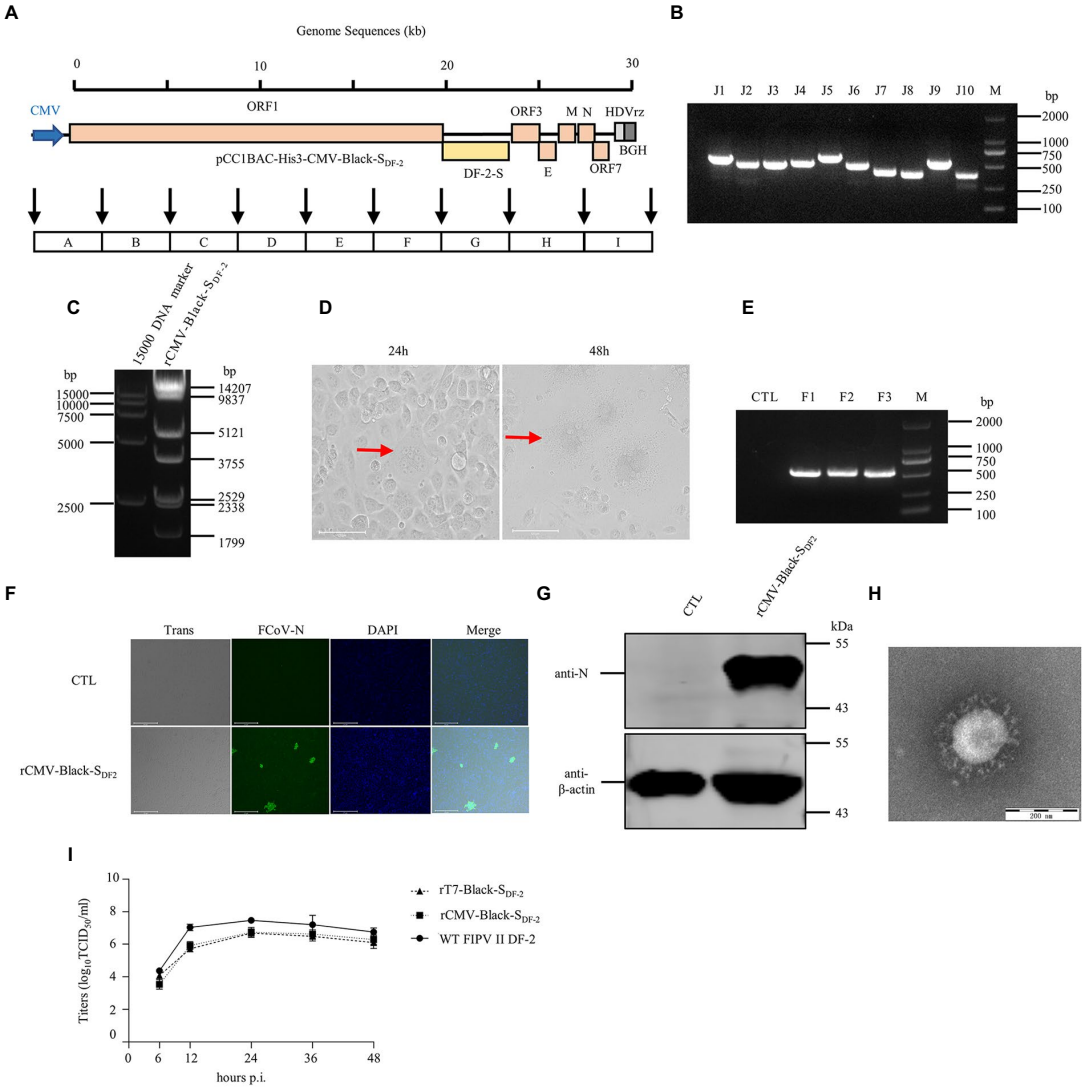
The recovery and identification of the chimeric FIPV strain Black-S_DF-2_ driven by a CMV promoter. **(A)** Strategy diagram for the construction of pCC1BAC-His3-CMV-Black-S_DF-2_. **(B)** Identification of fragment interfaces in bacteria. **(C)** Enzyme digestion of pCC1BAC-His3-CMV-Black-S_DF-2_ plasmid (*Not*I and *Nhe*I). **(D)** Significant cytopathic effects (CPE) were observed at 24 and 48 h after infection with CRFK cells. **(E)** PCR identification of the recombinant virus. **(F)** and **(G)** rCMV-Black-S_DF-2_ virus infected CRFK cells for 12 h was identified by IFA (Scale Bar, 275 μm) and WB. **(H)** Electron microscopy examination of the purified virus. **(I)** Growth kinetics of rT7-Black-S_DF-2_, rCMV-Black-S_DF-2_, and WT FIPV II DF-2 after infection of CRFK cells at an MOI of 0.01. wt, wild type.

**Figure 4:**
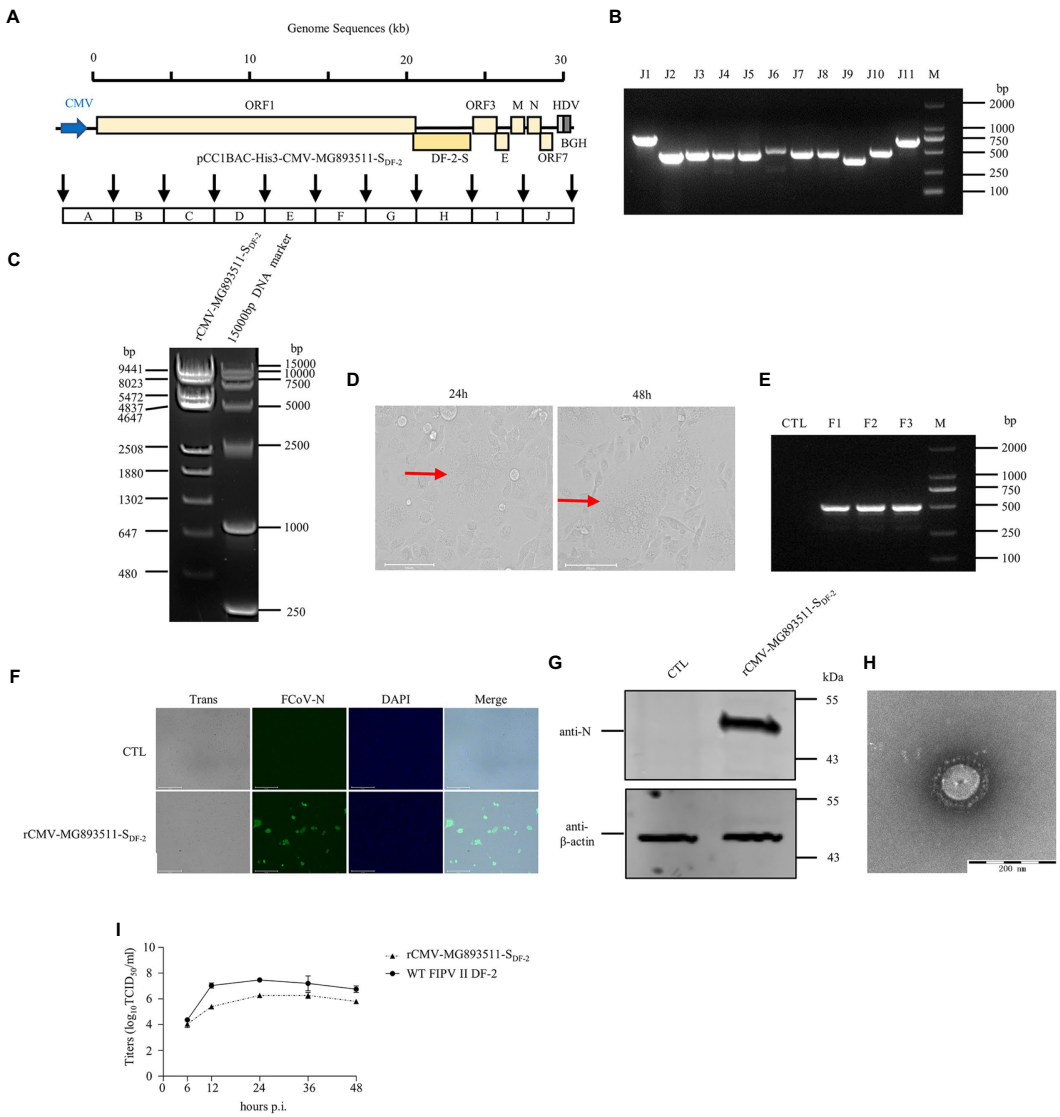
The recovery and identification of the chimeric FECV strain of MG893511-S_DF-2_. **(A)** Strategy diagram for the construction of pCC1BAC-His3-CMV-Black-S_DF-2_. **(B)** Identification of fragment interfaces of the plasmids recovered from bacteria. **(C)** Enzyme digestion of pCC1BAC-His3-CMV-MG893511-S_DF-2_ plasmid (*Bgl*II). **(D)** Significant cytopathic effects (CPE) were observed at 24 and 48 h after infection with CRFK cells. **(E)** PCR identification of the rescued recombinant FCoV virus. **(F)** and **(G)** rCMV-MG893511-S_DF-2_ virus infected CRFK cells for 12 h was identified by IFA (Scale Bar, 275 μm) and WB. **(H)** Electron microscopy of the purified virus. **(I)** Growth kinetics of rCMV-MG893511-S_DF-2_ and WT FIPV II DF-2 after infection of CRFK cells at an MOI of 0.01. wt, wild type.

**Figure 5:**
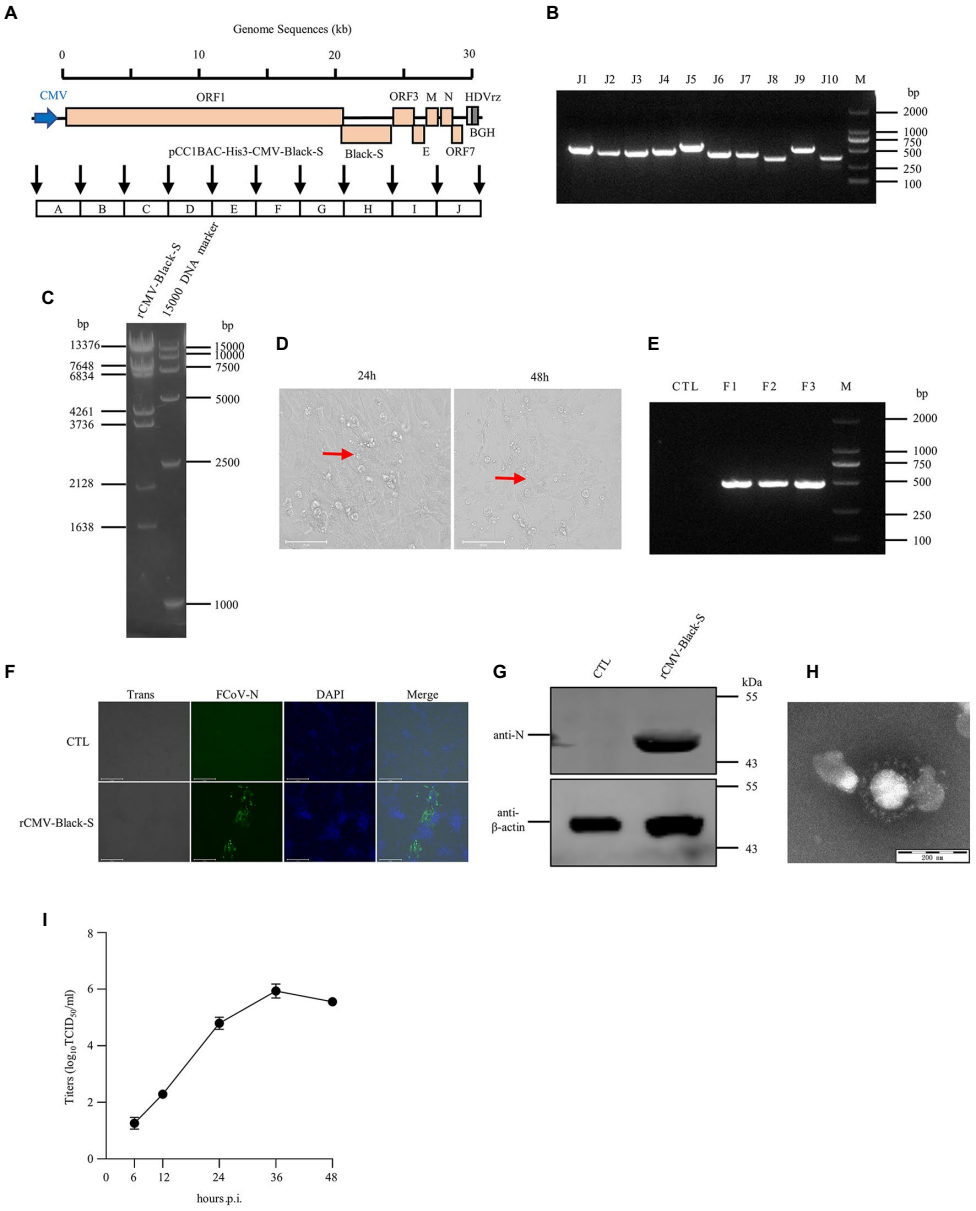
The recovery and identification of culture-adapted wild-type I FIPV Black strain. **(A)** Strategy diagram for the construction of pCC1BAC-His3-CMV-Black-S. **(B)** Identification of fragment interfaces of the plasmid recovered from bacteria. **(C)** Restriction enzyme digestion of the positive plasmid (*Nco*I) purified from *E. coli.* **(D)** Significant cytopathic effects (CPE) were observed at 24 and 48 h after infection with FCWF-4 cells. **(E)** PCR identification of the rescued virus. **(F)** and **(G)** rCMV-Black-S virus infected FCWF-4 cells for 12 h was identified by IFA (Scale Bar, 275 μm) and WB. Electron microscopy of the purified virus. **(H)** Electron microscopy examination of the purified virus. **(I)** Growth curve of rescued rCMV-Black-S virus on FCWF-4 cells with original MOI of 0.1.

A rescued virus harboring a CMV promoter would not need to be transcribed *in vitro* or assisted by T7 pol expression cell lines, which would make rescuing the virus faster and more convenient. Thus, we replaced the T7 promoter with the CMV promoter in the pCC1BAC-His3 as described above and used it to assemble several FCoV genomes. The strategy for constructing these clones in the pCC1BAC-His3 vector were shown in the Figure 3A (FIPV Black-S_DF-2_), Figure 4A (FECV MG893511-S_DF-2_) and Figure 5A (FIPV Black wild type). Correct assembly of the viral genome were screened and identified by PCR (Figure 3B: FIPV Black-S_DF-2_; Figure 4B: FECV MG893511-S_DF-2_; Figure 5B: FIPV Black wild type) and restriction enzyme analyses (Figure 3C: FIPV Black-S_DF-2_; Figure 4C: FECV MG893511-S_DF-2_; Figure 5C: FIPV Black wild type). The plasmids were then transfected into CRFK cells using PEI transfection reagent. After 48 h, the cells were freeze-thawed once. The resulting supernatant was placed on CRFK cells and cultured for 24 h. Cytopathic effects could be observed for all the three viruses (Figure 3D: FIPV Black-S_DF-2_; Figure 4D: FECV MG893511-S_DF-2_; Figure 5D: FIPV Black wild type). The rescued virus was further identified by PCR (Figure 3E: FIPV Black-S_DF-2_; Figure 4E: FECV MG893511-S_DF-2_; Figure 5E: FIPV Black wild type), western blotting (Figure 3F: FIPV Black-S_DF-2_; Figure 4F: FECV MG893511-S_DF-2_; Figure 5F: FIPV Black wild type), IFAT (Figure 3G: FIPV Black-S_DF-2_; Figure 4G: FECV MG893511-S_DF-2_; Figure 5G: FIPV Black wild type), and electron microscopy (Figure 3H: FIPV Black-S_DF-2_; Figure 4H: FECV MG893511-S_DF-2_; Figure 5H: FIPV Black wild type). All recombinant viruses with serotype II S protein could replicate properly, the rescued viruses displayed growth characteristics similar to those displayed by serotype II FCoV strain DF2 and reached peak titers at 24 h after infection (Figure 3I: FIPV Black-S_DF-2_; Figure 4I: FECV MG893511-S_DF-2_). There is no significant difference in the titer of rescued viruses with either a T7 promoter or a CMV promoter. The recombinant FIPV Black with wild type S protein could replicate in FCWF-4 cells with high MOI which was similar to previous study as shown in Figure 5I (Tekes et al., 2008).

## 4. Discussion

The emergence of FIPV poses a serious global threat to cat health. Consequently, research is required to improve our understanding of the biology and pathogenesis of this virus, and this should provide a basis for the development of intervention strategies. Currently, there is no definitive test to diagnose FIP. Although antibody levels or titers of coronaviruses can be measured, they cannot clearly distinguish FECV from FIPV. A positive result simply means the cat has previously been exposed to coronavirus but not necessarily to FIPV. FCoV infection usually causes mild or asymptomatic infection in cats, but mutants with increased pathogenicity may emerge during infection (Chang et al., 2011; Kipar and Meli, 2014; Pedersen, 2014; Tekes and Thiel, 2016). It is estimated that FIPV conversion occurs in about 5% of cats persistently infected with FECV (Chang et al., 2011; Felten and Hartmann, 2019). FIPV is thought to be a mutation of FECV that is prevalent in individual cats (Vennema et al., 1998). However, the nature of the mutation that results in the switch from FECV to FIPV is currently unknown (Kipar and Meli, 2014). To address this core problem, a reverse genetics system for both highly and weakly virulent FCoV is urgently needed.

The yeast-based TAR system has been used to construct coronaviruses (Thao et al., 2020). The system could further improve because the large quantities of yeast extraction is costly and complex, it also requires *in vitro* transcription, and the effect of RNA electrotransfer is not as good as that of DNA transfection. In this study, we assembled infective cDNA clones of FCoV using a pCC1BAC-His3 vector and amplified them through a copy-controlled system in *E. coli*. In addition, T7 promoter was replaced by CMV promoter which does not require *in vitro* transcription, and thus the experimental process is short and convenient. Consistent with previous reports, without the need for tedious cloning processes, the construction of infective cDNA of FCoV could be completed in 1 week.

Type I FCoV causes about 80% of the natural infections of cats worldwide, while the other 20% are due to type II FCoV. To date, most research on FCoV has focused on type II viruses, mainly because they can be easily grown in cell culture compared to type I FCoV. Here, a culture-adapted type I Black strain was also rescued through this system. The type I Black strain is one of the few strains that can grow in cells. Although we have not introduced Black strain successfully due to the COVID-19 epidemic, but the recombinant FIPV Black with wild type S protein in our study displayed growth characteristics similar to previous study (Tekes et al., 2008). The successful establishment of reverse genetics of this strain based on our TAR system will further facilitate the study of the pathogenesis of type I FCoV. In addition, the S gene used in the previous reverse genetic systems was derived from the type II strain 79–1,146. The author found that substituting S gene of this strain could not induce biotype conversion of avirulent viruses to pathogenic ones. Therefore, it will be interesting to evaluate the pathogenesis of a virus carrying a S gene from a different type II FIPV, for example, the viruses carrying S gene of DF II strain rescued in this study.

The ability to systematically exchange genome fragments from FECV with matching fragments from FIPV, as described in this study, will enable mapping of the genetic changes required to convert a FECV biotype into a FIPV biotype. Such a reverse genetic system would be helpful not only in the study of culture-adapted coronaviruses but also in that of wild-type I coronaviruses, for which there are currently no suitable cell culture systems. In conclusion, the virulent and attenuated full-length cDNA clones of FCoV described here provide a powerful tool for studying the transmission and pathogenesis of FCoV, and evaluating FIP vaccines and therapeutics.

## Data availability statement

The original contributions presented in the study are included in the article/Supplementary material, further inquiries can be directed to the corresponding authors.

## Author contributions

HK and HJ: conceptualization. HC and HG: investigation. HK and HC: original draft preparation. HK and HJ: review and editing. All authors have read and agreed to publish the version of manuscript. Author order was determined based on workload.

## Conflict of interest

The authors declare that the research was conducted in the absence of any commercial or financial relationships that could be construed as a potential conflict of interest.

## Publisher’s note

All claims expressed in this article are solely those of the authors and do not necessarily represent those of their affiliated organizations, or those of the publisher, the editors and the reviewers. Any product that may be evaluated in this article, or claim that may be made by its manufacturer, is not guaranteed or endorsed by the publisher.
