# Supplementary material for: Development of a rapid reverse genetics system for feline coronavirus based on TAR cloning in yeast
Source: Front Microbiol. 2023 Mar 23;14:1141101. doi: 10.3389/fmicb.2023.1141101 (PMC10076789; doi:10.3389/fmicb.2023.1141101)
Supplement: Supplementary file 1 [file Table_1.DOCX]

Supplementary Material

Development of a novel rapid reverse genetics system for Feline Coronavirus

**Hongmin Cao ^1†^, Haorong Gu ^1†^, Hongtao Kang ^1^*, and Honglin Jia ^1^***

*** Correspondence:** [kanghongtao@caas.cn](mailto:kanghongtao@caas.cn) (H.K.); [jiahonglin@caas.cn](mailto:jiahonglin@caas.cn) (H.J.)

† These authors contributed equally to this work. Author order was determined based on workload.

**Supplementary Data**

## Supplementary TABLES

**TABLE S1.** Amplification primers used in this study.

| Plasmid | Name of amplified fragment | Primer name | Primer sequence (5'-3') | Amplification product (bp) |
| --- | --- | --- | --- | --- |
| pCC1BAC-His3-T7-Black-S_DF-2_ | A | PCC1BAC-HIS3-3511-1-F(△PMEI) | TGTAAAACGACGGCCAGTGAATAATACGACTCACTATAGACTTTTAAAGTAAAGTGAG | 6196 |
| pCC1BAC-His3-T7-Black-S_DF-2_ |  | BlacK-2-R | CTTTACAAAACTAGAATTACTATATGCTTGAACACTACCACTACATGCCAAT |  |
| pCC1BAC-His3-T7-Black-S_DF-2_ | B | Black-3-f | CCAGTAGTGCATAAATTG | 6097 |
| pCC1BAC-His3-T7-Black-S_DF-2_ |  | Black-4R | GTTCTACATGACATCTACAATAAATGCATACAGATGCGCCACCATAAGAG |  |
| pCC1BAC-His3-T7-Black-S_DF-2_ | C | Black-5F | CACACAACAGGACTCTTATG | 6052 |
| pCC1BAC-His3-T7-Black-S_DF-2_ |  | Black-6-R | GGTTATTAACATACAATGCACCACCATTACAACCTTCTAATGACAGTTTTG |  |
| pCC1BAC-His3-T7-Black-S_DF-2_ | D | BLACK-7F | GATACTCGAACCCGCTCAAAAC | 2326 |
| pCC1BAC-His3-T7-Black-S_DF-2_ |  | Black-7R | CATAACAACAAGAGGCAAGTTACGAGCACAATCATGGTGTGTTAACAAAG |  |
| pCC1BAC-His3-T7-Black-S_DF-2_ | E | BLACK-7-DF2-S-F | GTTACTAAACTTTGGTAATCACTTTGTTAACACACCATGATTGTGCTCGTAACTTG | 4439 |
| pCC1BAC-His3-T7-Black-S_DF-2_ |  | DF2-S-Black-ORF3-R | GATGCCAATAGACTTGACGGTGTCCATAAATCGTTTAGTTAGTGGACATGCACTTTTTC |  |
| pCC1BAC-His3-T7-Black-S_DF-2_ | F | DF2-F-Black-ORF3-F | GAACCAATTGAAAAAGTGCATGTCCACTAACTAAACGATTTATGGACACCGTCAAGTCT | 1099 |
| pCC1BAC-His3-T7-Black-S_DF-2_ |  | BLACK-ORF3-E-R | GCCATGGTCATCTATGATGGTAAATGCCCTAGGAAACATCATAGACACGC |  |
| pCC1BAC-His3-T7-Black-S_DF-2_ | G | BLACK-ORF3-E-F | CGAAATTGATTTGAAAGAAGAAGAAGAGCGTGTCTATGATGTTTCCTAGGGCATTTAC | 3479 |
| pCC1BAC-His3-T7-Black-S_DF-2_ |  | BLACK-A-R-HDV | GCCATGCCGACCCTTTTTTTTTTTTTTTTTTTTTTTTTTTGTGTATCACTATCAAAAGGAAAATTTTC |  |
| pCC1BAC-His3-T7-Black-S_DF-2_ | H | BLACK-HDV-F | TTTTCCTTTTGATAGTGATACACAAAAAAAAAAAAAAAAAAAAAAAAAAAGGGTCGGCATGGCATCTC | 399 |
| pCC1BAC-His3-T7-Black-S_DF-2_ |  | BGH-PCC-R | AAGCTTGCATGCCTGCAGGTCGACTCTAGAGAATTCCCATAGAGCCCACCGCATC |  |
| pCC1BAC-His3-CMV-Black-S_DF-2_ | A | pCC1BAC-His3-CMV-F | AGTCACGACGTTGTAAAACGACGGCCAGTGAAGTTGACATTGATTATTGACTAG | 653 |
| pCC1BAC-His3-CMV-Black-S_DF-2_ |  | CMV-BLACK-R | TATAGCCCAGCTACACTCACTTTACTTTAAAAGTAGCTCTGCTTATATAGACCTCC |  |
| pCC1BAC-His3-CMV-Black-S_DF-2_ | B | CMV-FECV-F | GTGTACGGTGGGAGGTCTATATAAGCAGAGCTACTTTTAAAGTAAAGTGAGTGTAG | 6190 |
| pCC1BAC-His3-CMV-Black-S_DF-2_ |  | BlacK-2-R | CTTTACAAAACTAGAATTACTATATGCTTGAACACTACCACTACATGCCAAT |  |
| pCC1BAC-His3-CMV-Black-S_DF-2_ | C | Black-3-f | CCAGTAGTGCATAAATTG | 6097 |
| pCC1BAC-His3-CMV-Black-S_DF-2_ |  | Black-4R | GTTCTACATGACATCTACAATAAATGCATACAGATGCGCCACCATAAGAG |  |
| pCC1BAC-His3-CMV-Black-S_DF-2_ | D | Black-5F | CACACAACAGGACTCTTATG | 6052 |
| pCC1BAC-His3-CMV-Black-S_DF-2_ |  | Black-6-R | GGTTATTAACATACAATGCACCACCATTACAACCTTCTAATGACAGTTTTG |  |
| pCC1BAC-His3-CMV-Black-S_DF-2_ | E | BLACK-7F | GATACTCGAACCCGCTCAAAAC | 2326 |
| pCC1BAC-His3-CMV-Black-S_DF-2_ |  | Black-7R | CATAACAACAAGAGGCAAGTTACGAGCACAATCATGGTGTGTTAACAAAG |  |
| pCC1BAC-His3-CMV-Black-S_DF-2_ | F | BLACK-7-DF2-S-F | GTTACTAAACTTTGGTAATCACTTTGTTAACACACCATGATTGTGCTCGTAACTTG | 4439 |
| pCC1BAC-His3-CMV-Black-S_DF-2_ |  | DF2-S-Black-ORF3-R | GATGCCAATAGACTTGACGGTGTCCATAAATCGTTTAGTTAGTGGACATGCACTTTTTC |  |
| pCC1BAC-His3-CMV-Black-S_DF-2_ | G | DF2-F-Black-ORF3-F | GAACCAATTGAAAAAGTGCATGTCCACTAACTAAACGATTTATGGACACCGTCAAGTCT | 1099 |
| pCC1BAC-His3-CMV-Black-S_DF-2_ |  | BLACK-ORF3-E-R | GCCATGGTCATCTATGATGGTAAATGCCCTAGGAAACATCATAGACACGC |  |
| pCC1BAC-His3-CMV-Black-S_DF-2_ | H | BLACK-ORF3-E-F | CGAAATTGATTTGAAAGAAGAAGAAGAGCGTGTCTATGATGTTTCCTAGGGCATTTAC | 3479 |
| pCC1BAC-His3-CMV-Black-S_DF-2_ |  | BLACK-A-R-HDV | GCCATGCCGACCCTTTTTTTTTTTTTTTTTTTTTTTTTTTGTGTATCACTATCAAAAGGAAAATTTTC |  |
| pCC1BAC-His3-CMV-Black-S_DF-2_ | I | BLACK-HDV-F | TTTTCCTTTTGATAGTGATACACAAAAAAAAAAAAAAAAAAAAAAAAAAAGGGTCGGCATGGCATCTC | 399 |
| pCC1BAC-His3-CMV-Black-S_DF-2_ |  | BGH-PCC-R | AAGCTTGCATGCCTGCAGGTCGACTCTAGAGAATTCCCATAGAGCCCACCGCATC |  |
| pCC1BAC-His3-CMV-MG893511-S_DF-2_ | A | pCC1BAC-His3-CMV-F | AGTCACGACGTTGTAAAACGACGGCCAGTGAAGTTGACATTGATTATTGACTAG | 653 |
| pCC1BAC-His3-CMV-MG893511-S_DF-2_ |  | CMV-R | TATAGCCACGCTACACTCACTTTACTTTAAAAGTAGCTCTGCTTATATAGACCTCC |  |
| pCC1BAC-His3-CMV-MG893511-S_DF-2_ | B | CMV-FECV-F | GTGTACGGTGGGAGGTCTATATAAGCAGAGCTACTTTTAAAGTAAAGTGAGTGTAG | 3053 |
| pCC1BAC-His3-CMV-MG893511-S_DF-2_ |  | FECV-1-R | CTAGCTTAACACGTGTAACAGGTTCTATCTCTCTAACATTAACA |  |
| pCC1BAC-His3-CMV-MG893511-S_DF-2_ | C | 2-F | TGACAAGTCGGTTTCTTTTTCAGATAATGTTAATGTTAGAGAGATAGAACCTGTTACACGTGTTAAGC | 3100 |
| pCC1BAC-His3-CMV-MG893511-S_DF-2_ |  | 2-R | TAGTTTTATGTAACGCCACAATGCTGGTTTGTTCAATTGTCTTACATAATTCAATGTTCTAACTTTAG |  |
| pCC1BAC-His3-CMV-MG893511-S_DF-2_ | D | 3-F | CTAAAGTTAGAACATTGAATTATGTAAGACAATTGA | 3036 |
| pCC1BAC-His3-CMV-MG893511-S_DF-2_ |  | 3-R | GTGCCATTTTTCGCAGTCCAGACTGTAGTGTTGAGT |  |
| pCC1BAC-His3-CMV-MG893511-S_DF-2_ | E | 4-F | CATGCTTTACACGCCACCGACCGTTAGTGTTAACTCAACACTACAGTCTGGACTGCGAAAAATGGCAC | 3100 |
| pCC1BAC-His3-CMV-MG893511-S_DF-2_ |  | 4-R | TCGTTTAACAGCATCAACATATGCTTTAGCAGGGTCAGGTGCAAAAGCACATAGAGTCAGCAAACCAC |  |
| pCC1BAC-His3-CMV-MG893511-S_DF-2_ | F | 5-F | GTGGTTTGCTGACTCTATGTGCTTTTGCACCTGACC | 3036 |
| pCC1BAC-His3-CMV-MG893511-S_DF-2_ |  | 5-R | AAATGTTTAACCCAATCCAGAAGAGCATAAAATACT |  |
| pCC1BAC-His3-CMV-MG893511-S_DF-2_ | G | 6-F | ACTCACAAAACACCCTAAACCTGCATACCAAAGAGTATTTTATGCTCTTCTGGATTGGGTTAAACATTT | 3101 |
| pCC1BAC-His3-CMV-MG893511-S_DF-2_ |  | 6-R | TTTGTCCATGTACCATATAATCATACTCCAGACATCTAACATTGTTATTAATAGGATCACGGTCATAA |  |
| pCC1BAC-His3-CMV-MG893511-S_DF-2_ | H | 7-F | TTATGACCGTGATCCTATTAATAACAATGTTAGATG | 2448 |
| pCC1BAC-His3-CMV-MG893511-S_DF-2_ |  | 7-R | AGTTACGAGCACAATCATGGTGTATTAACAAAGTGA |  |
| pCC1BAC-His3-CMV-MG893511-S_DF-2_ | I | 7+S-F | AGAAACAATGGTAAGTTACTAAACTTTGGTAATCACTTTGTTAATACACCATGATTGTGCTCGTAACT | 4465 |
| pCC1BAC-His3-CMV-MG893511-S_DF-2_ |  | S+8-R | GGCGTCAACAGAGAAGCTTATAGACTTGACAGTGTCCATAAATCGTTTAGTTAGTGGACATGCACTTT |  |
| pCC1BAC-His3-CMV-MG893511-S_DF-2_ | G | 8-F | AAAGTGCATGTCCACTAACTAAACGATTTATGGACA | 3036 |
| pCC1BAC-His3-CMV-MG893511-S_DF-2_ |  | 8-R | TCAGCAGACCATTGACTGCCGAAGAGTATGCTAGAT |  |
| pCC1BAC-His3-CMV-MG893511-S_DF-2_ | K | 9-F | TACCCTCAGATAGCTGAATGCGTTCCATCAGTATCTAGCATACTCTTCGGCAGTCAATGGTCTGCTGA | 1914 |
| pCC1BAC-His3-CMV-MG893511-S_DF-2_ |  | BGH-PCC-R | AAGCTTGCATGCCTGCAGGTCGACTCTAGAGAATTCCCATAGAGCCCACCGCATC |  |
| pCC1BAC-His3-CMV-Black-S | A | pCC1BAC-His3-CMV-F | AGTCACGACGTTGTAAAACGACGGCCAGTGAAGTTGACATTGATTATTGACTAG | 653 |
| pCC1BAC-His3-CMV-Black-S |  | CMV-BLACK-R | TATAGCCCAGCTACACTCACTTTACTTTAAAAGTAGCTCTGCTTATATAGACCTCC |  |
| pCC1BAC-His3-CMV-Black-S | B | CMV-FECV-F | GTGTACGGTGGGAGGTCTATATAAGCAGAGCTACTTTTAAAGTAAAGTGAGTGTAG | 6190 |
| pCC1BAC-His3-CMV-Black-S |  | BlacK-2-R | CTTTACAAAACTAGAATTACTATATGCTTGAACACTACCACTACATGCCAAT |  |
| pCC1BAC-His3-CMV-Black-S | C | Black-3-f | CCAGTAGTGCATAAATTG | 6097 |
| pCC1BAC-His3-CMV-Black-S |  | Black-4R | GTTCTACATGACATCTACAATAAATGCATACAGATGCGCCACCATAAGAG |  |
| pCC1BAC-His3-CMV-Black-S | D | Black-5F | CACACAACAGGACTCTTATG | 6052 |
| pCC1BAC-His3-CMV-Black-S |  | Black-6-R | GGTTATTAACATACAATGCACCACCATTACAACCTTCTAATGACAGTTTTG |  |
| pCC1BAC-His3-CMV-Black-S | E | BLACK-7F | GATACTCGAACCCGCTCAAAAC | 2326 |
| pCC1BAC-His3-CMV-Black-S |  | BLACK-7-S-R | GGAGTGCAAATATTAACACTATCATGGTGTGTTAACAAAGTGATTACC |  |
| pCC1BAC-His3-CMV-Black-S | F | BLACK-1B-S-F | AACTTTGGTAATCACTTTGTTAACACACCATGATAGTGTTAATATTTGCACTCC | 4395 |
| pCC1BAC-His3-CMV-Black-S |  | BLACK-S-ORF3-R | ATAGACTTGACGGTGTCCATAAATCGTTTAGTTAGTGAATGTGAACCTTTTCAATAG |  |
| pCC1BAC-His3-CMV-Black-S | G | BLACK-S-ORF3-F | ATTGAAAAGGTTCACATTCACTAACTAAACGATTTATGGACACCGTCAAGTCTATTG | 1099 |
| pCC1BAC-His3-CMV-Black-S |  | BLACK-ORF3-E-R | GCCATGGTCATCTATGATGGTAAATGCCCTAGGAAACATCATAGACACGC |  |
| pCC1BAC-His3-CMV-Black-S | H | BLACK-ORF3-E-F | CGAAATTGATTTGAAAGAAGAAGAAGAGCGTGTCTATGATGTTTCCTAGGGCATTTAC | 3479 |
| pCC1BAC-His3-CMV-Black-S |  | BLACK-A-R-HDV | GCCATGCCGACCCTTTTTTTTTTTTTTTTTTTTTTTTTTTGTGTATCACTATCAAAAGGAAAATTTTC |  |
| pCC1BAC-His3-CMV-Black-S | I | BLACK-HDV-F | TTTTCCTTTTGATAGTGATACACAAAAAAAAAAAAAAAAAAAAAAAAAAAGGGTCGGCATGGCATCTC | 399 |
| pCC1BAC-His3-CMV-Black-S |  | BGH-PCC-R | AAGCTTGCATGCCTGCAGGTCGACTCTAGAGAATTCCCATAGAGCCCACCGCATC |  |

TABLE S2. Identification primers used in this study.

| Plasmid | Primer name | Primer sequence(5'-3') | Amplification product (bp) |
| --- | --- | --- | --- |
| pCC1BAC-His3-T7-Black-S_DF-2_ | pCC1BAC-His3-JD-F | CTGAGAGTGCACCATATGC | 449 |
| pCC1BAC-His3-T7-Black-S_DF-2_ | MG893511-1+604JD-R | CTTACCAAACAGGGGCGGA |  |
| pCC1BAC-His3-T7-Black-S_DF-2_ | Black-JD-2F | GCTAGCTATTGAGAGTGGTG | 505 |
| pCC1BAC-His3-T7-Black-S_DF-2_ | BLACK-JD-3R | CTATTCCATAGTGGGTCTGAC |  |
| pCC1BAC-His3-T7-Black-S_DF-2_ | BLACK-JD-4F | CACTCTTAGACGTGGTGC | 520 |
| pCC1BAC-His3-T7-Black-S_DF-2_ | BLACK-JD-5R | CTAGTCGAGCTGCACTAG |  |
| pCC1BAC-His3-T7-Black-S_DF-2_ | BLACK-JD-6F | GCTGGTCGCATTGTACAATC | 613 |
| pCC1BAC-His3-T7-Black-S_DF-2_ | BLACK-JD-7R | CTTGCCACAACATGTAAGTGTC |  |
| pCC1BAC-His3-T7-Black-S_DF-2_ | BLACK-7-DF2S-jiandingF | CTGTGACAAGGCTATAGTGGATGG | 486 |
| pCC1BAC-His3-T7-Black-S_DF-2_ | DF2-S-jiandingR | GGCAGTAGTTTGTGCTGTTCT |  |
| pCC1BAC-His3-T7-Black-S_DF-2_ | BLACK-JD-DF2-F | CTTGCCATTCTCATTGATACC | 481 |
| pCC1BAC-His3-T7-Black-S_DF-2_ | Black-JD-ORF3-R | GTCGTATGATTAGGCGACTCTTC |  |
| pCC1BAC-His3-T7-Black-S_DF-2_ | Black-JD-ORF3-F | CCTATGCTTGTAGGCATAGC | 372 |
| pCC1BAC-His3-T7-Black-S_DF-2_ | Black-JD-3400-R | GCATGGCGTGCAGGTAGT |  |
| pCC1BAC-His3-T7-Black-S_DF-2_ | BLACK-JD-3400-F | CTGAGTAAGGCAACCCGATG | 530 |
| pCC1BAC-His3-T7-Black-S_DF-2_ | BGH-PCC-JD-R | GACACCTACTCAGACAATGCG |  |
| pCC1BAC-His3-T7-Black-S_DF-2_ | FECV-HDV-JD-F | CGACCTGGGCATCCGAAG | 375 |
| pCC1BAC-His3-T7-Black-S_DF-2_ | M13R | CAGGAAACAGCTATGAC |  |
| pCC1BAC-His3-CMV-Black-S_DF-2_ | pCC1BAC-His3-JD-F | CTGAGAGTGCACCATATGC | 587 |
| pCC1BAC-His3-CMV-Black-S_DF-2_ | PCC-CMV-JD-R | CGTAGATGTACTGCCAAGTAGG |  |
| pCC1BAC-His3-CMV-Black-S_DF-2_ | CMV-FECV-1-JD-F | CCTGGCATTATGCCCAGTAC | 509 |
| pCC1BAC-His3-CMV-Black-S_DF-2_ | MG893511-1+604JD-R | CTTACCAAACAGGGGCGGA |  |
| pCC1BAC-His3-CMV-Black-S_DF-2_ | Black-JD-2F | GCTAGCTATTGAGAGTGGTG | 505 |
| pCC1BAC-His3-CMV-Black-S_DF-2_ | BLACK-JD-3R | CTATTCCATAGTGGGTCTGAC |  |
| pCC1BAC-His3-CMV-Black-S_DF-2_ | BLACK-JD-4F | CACTCTTAGACGTGGTGC | 520 |
| pCC1BAC-His3-CMV-Black-S_DF-2_ | BLACK-JD-5R | CTAGTCGAGCTGCACTAG |  |
| pCC1BAC-His3-CMV-Black-S_DF-2_ | BLACK-JD-6F | GCTGGTCGCATTGTACAATC | 613 |
| pCC1BAC-His3-CMV-Black-S_DF-2_ | BLACK-JD-7R | CTTGCCACAACATGTAAGTGTC |  |
| pCC1BAC-His3-CMV-Black-S_DF-2_ | BLACK-7-DF2S-jiandingF | CTGTGACAAGGCTATAGTGGATGG | 486 |
| pCC1BAC-His3-CMV-Black-S_DF-2_ | DF2-S-jiandingR | GGCAGTAGTTTGTGCTGTTCT |  |
| pCC1BAC-His3-CMV-Black-S_DF-2_ | BLACK-JD-DF2-F | CTTGCCATTCTCATTGATACC | 481 |
| pCC1BAC-His3-CMV-Black-S_DF-2_ | Black-JD-ORF3-R | GTCGTATGATTAGGCGACTCTTC |  |
| pCC1BAC-His3-CMV-Black-S_DF-2_ | Black-JD-ORF3-F | CCTATGCTTGTAGGCATAGC | 372 |
| pCC1BAC-His3-CMV-Black-S_DF-2_ | Black-JD-3400-R | GCATGGCGTGCAGGTAGT |  |
| pCC1BAC-His3-CMV-Black-S_DF-2_ | BLACK-JD-3400-F | CTGAGTAAGGCAACCCGATG | 530 |
| pCC1BAC-His3-CMV-Black-S_DF-2_ | BGH-PCC-JD-R | GACACCTACTCAGACAATGCG |  |
| pCC1BAC-His3-CMV-Black-S_DF-2_ | FECV-HDV-JD-F | CGACCTGGGCATCCGAAG | 375 |
| pCC1BAC-His3-CMV-Black-S_DF-2_ | M13R | CAGGAAACAGCTATGAC |  |
| pCC1BAC-His3-CMV-MG893511-S_DF-2_ | M13 fwd | AAAACGACGGCCAGT | 828 |
| pCC1BAC-His3-CMV-MG893511-S_DF-2_ | MG893511-1+604JD-R | CTTACCAAACAGGGGCGGA |  |
| pCC1BAC-His3-CMV-MG893511-S_DF-2_ | MG893511-1+2JD-F | GCCTGTAACTAATAGTGGCACTG | 501 |
| pCC1BAC-His3-CMV-MG893511-S_DF-2_ | MG893511-1+2JD-R | GAAATCATAACACCGTCTGGGTTG |  |
| pCC1BAC-His3-CMV-MG893511-S_DF-2_ | MG893511-2+3JD-F | CCTCTTACAGGTTACAACAGCCA | 516 |
| pCC1BAC-His3-CMV-MG893511-S_DF-2_ | MG893511-2+3JD-R | GCTTGAACACTACCACTACATGCC |  |
| pCC1BAC-His3-CMV-MG893511-S_DF-2_ | MG893511-3+4JD-F | GAATCAGCCGCTATGGGTAC | 507 |
| pCC1BAC-His3-CMV-MG893511-S_DF-2_ | MG893511-3+4JD-R | CACACCCTTGTATTTGGCAG |  |
| pCC1BAC-His3-CMV-MG893511-S_DF-2_ | MG893511-4+5JD-F | GCATTTATAGCATCAGACAGCAACC | 488 |
| pCC1BAC-His3-CMV-MG893511-S_DF-2_ | MG893511-4+5JD-R | CCTTTAAAGCGGCACAAACCATC |  |
| pCC1BAC-His3-CMV-MG893511-S_DF-2_ | MG893511-5+6JD-F | GTGTTGGGTGGAACCAGATC | 556 |
| pCC1BAC-His3-CMV-MG893511-S_DF-2_ | MG893511-5+6JD-R | GCTACACACATACGGTGTGATAGAC |  |
| pCC1BAC-His3-CMV-MG893511-S_DF-2_ | MG893511-6+7JD-F | CGCTATCATGACTAGGTGTCTTGC | 496 |
| pCC1BAC-His3-CMV-MG893511-S_DF-2_ | MG893511-6+7JD-R | GAATGGCATAGGTTTAAGCTTAGC |  |
| pCC1BAC-His3-CMV-MG893511-S_DF-2_ | BLACK-7-DF2S-jiandingF | CTGTGACAAGGCTATAGTGGATGG | 486 |
| pCC1BAC-His3-CMV-MG893511-S_DF-2_ | DF2-S-jiandingR | GGCAGTAGTTTGTGCTGTTCT |  |
| pCC1BAC-His3-CMV-MG893511-S_DF-2_ | DF2-S-ORF3 jd F | GGCCTTGGTATGTGTGGCTAC | 391 |
| pCC1BAC-His3-CMV-MG893511-S_DF-2_ | DF2-S orf3 jd-R | AGACGGCTCTTCAAAATCCAAC |  |
| pCC1BAC-His3-CMV-MG893511-S_DF-2_ | MG893511-8+9JD-F | GGTCACGTTCTAGATCTGGAG | 504 |
| pCC1BAC-His3-CMV-MG893511-S_DF-2_ | MG893511-8+9JD-R | CCTCATCAATCATCTCAACCTG |  |
| pCC1BAC-His3-CMV-MG893511-S_DF-2_ | MG893511-9+604JD-F | CCGCTATGACGAGCCAACAATGG | 567 |
| pCC1BAC-His3-CMV-MG893511-S_DF-2_ | PCC1BAC-HIS3-JD-R | GCCGATTCATTAATGCAGCTG |  |
| pCC1BAC-His3-CMV-Black-S | pCC1BAC-His3-JD-F | CTGAGAGTGCACCATATGC | 587 |
| pCC1BAC-His3-CMV-Black-S | PCC-CMV-JD-R | CGTAGATGTACTGCCAAGTAGG |  |
| pCC1BAC-His3-CMV-Black-S | CMV-FECV-1-JD-F | CCTGGCATTATGCCCAGTAC | 509 |
| pCC1BAC-His3-CMV-Black-S | MG893511-1+604JD-R | CTTACCAAACAGGGGCGGA |  |
| pCC1BAC-His3-CMV-Black-S | Black-JD-2F | GCTAGCTATTGAGAGTGGTG | 505 |
| pCC1BAC-His3-CMV-Black-S | BLACK-JD-3R | CTATTCCATAGTGGGTCTGAC |  |
| pCC1BAC-His3-CMV-Black-S | BLACK-JD-4F | CACTCTTAGACGTGGTGC | 520 |
| pCC1BAC-His3-CMV-Black-S | BLACK-JD-5R | CTAGTCGAGCTGCACTAG |  |
| pCC1BAC-His3-CMV-Black-S | BLACK-JD-6F | GCTGGTCGCATTGTACAATC | 613 |
| pCC1BAC-His3-CMV-Black-S | BLACK-JD-7R | CTTGCCACAACATGTAAGTGTC |  |
| pCC1BAC-His3-CMV-Black-S | BLACK-7-DF2S-jiandingF | CTGTGACAAGGCTATAGTGGATGG | 453 |
| pCC1BAC-His3-CMV-Black-S | Black-JD-S-R | GTCTCCACAGTAGGGTAGG |  |
| pCC1BAC-His3-CMV-Black-S | Black-JD--S-F | CTCGAATGGCTCAACAGG | 439 |
| pCC1BAC-His3-CMV-Black-S | Black-JD-ORF3-R | GTCGTATGATTAGGCGACTCTTC |  |
| pCC1BAC-His3-CMV-Black-S | Black-JD-ORF3-F | CCTATGCTTGTAGGCATAGC | 372 |
| pCC1BAC-His3-CMV-Black-S | Black-JD-3400-R | GCATGGCGTGCAGGTAGT |  |
| pCC1BAC-His3-CMV-Black-S | BLACK-JD-3400-F | CTGAGTAAGGCAACCCGATG | 530 |
| pCC1BAC-His3-CMV-Black-S | BGH-PCC-JD-R | GACACCTACTCAGACAATGCG |  |
| pCC1BAC-His3-CMV-Black-S | FECV-HDV-JD-F | CGACCTGGGCATCCGAAG | 375 |
| pCC1BAC-His3-CMV-Black-S | M13R | CAGGAAACAGCTATGAC |  |

TABLE S3. The gene sequences used in the study.

| Gene name | Size | Sequence |
| --- | --- | --- |
| CEN6 | 117bp | ATCACGTGCTATAAAAATAATTATAATTTAAATTTTTTAATATAAATATATAAATTAAAAATAGAAAGTAAAAAAAGAAATTAAAGAAAAAATAGTTTTTGTTTTCCGAAGATGTAA |
| ARS | 388bp | AAGACTCTAGGGGGATCGCCAACAAATACTACCTTTTATCTTGCTCTTCCTGCTCTCAGGTATTAATGCCGAATTGTTTCATCTTGTCTGTGTAGAAGACCACACACGAAAATCCTGTGATTTTACATTTTACTTATCGTTAATCGAATGTATATCTATTTAATCTGCTTTTCTTGTCTAATAAATATATATGTAAAGTACGCTTTTTGTTGAAATTTTTTAAACCTTTGTTTATTTTTTTTTCTTCATTCCGTAACTCTTCTACCTTCTTTATTTACTTTCTAAAATCCAAATACAAAACATAAAAATAAATAAACACAGAGTAAATTCCCAAATTATTCCATCATTAAAAGATACGAGGCGCGTGTAAGTTACAGGCAAGCGATCC |
| His3 | 872bp | TAGTACACTCTATATTTTTTTATGCCTCGGTAATGATTTTCATTTTTTTTTTTCCACCTAGCGGATGACTCTTTTTTTTTCTTAGCGATTGGCATTATCACATAATGAATTATACATTATATAAAGTAATGTGATTTCTTCGAAGAATATACTAAAAAATGAGCAGGCAAGATAAACGAAGGCAAAGATGACAGAGCAGAAAGCCCTAGTAAAGCGTATTACAAATGAAACCAAGATTCAGATTGCGATCTCTTTAAAGGGTGGTCCCCTAGCGATAGAGCACTCGATCTTCCCAGAAAAAGAGGCAGAAGCAGTAGCAGAACAGGCCACACAATCGCAAGTGATTAACGTCCACACAGGTATAGGGTTTCTGGACCATATGATACATGCTCTGGCCAAGCATTCCGGCTGGTCGCTAATCGTTGAGTGCATTGGTGACTTACACATAGACGACCATCACACCACTGAAGACTGCGGGATTGCTCTCGGTCAAGCTTTTAAAGAGGCCCTACTGGCGCGTGGAGTAAAAAGGTTTGGATCAGGATTTGCGCCTTTGGATGAGGCACTTTCCAGAGCGGTGGTAGATCTTTCGAACAGGCCGTACGCAGTTGTCGAACTTGGTTTGCAAAGGGAGAAAGTAGGAGATCTCTCTTGCGAGATGATCCCGCATTTTCTTGAAAGCTTTGCAGAGGCTAGCAGAATTACCCTCCACGTTGATTGTCTGCGAGGCAAGAATGATCATCACCGTAGTGAGAGTGCGTTCAAGGCTCTTGCGGTTGCCATAAGAGAAGCCACCTCGCCCAATGGTACCAACGATGTTCCCTCCACCAAAGGTGTTCTTATGTAGTTTTACACAGGAGTCTGGACTTGAC |
